# Active treatment of patients with “incurable disease”: what’s the point?

**DOI:** 10.1007/s12682-012-0113-2

**Published:** 2012-03-09

**Authors:** A. Goisis

**Affiliations:** U.O. Cure Palliative-Hospice, Casa di Cura “Beato Palazzolo”, via San Bernardino, 56, 24122 Bergamo, Italy

**Keywords:** Death, Active treatment, Emotional suffering, Patient’s needs, Palliative care, Hope, Spirituality

## Abstract

What is active treatment of patients with “incurable disease”? I believe that it is not limited to the care of the patients’ symptoms, but goes further. We can and must control pain, breathlessness, nausea and vomiting, intestinal obstruction, insomnia… but something else remains for us to face, something paradoxically made more evident by the absence of symptoms: the patients’ emotional suffering, suffering deep in their heart, the most hidden, but which becomes apparent in the season of death. So, what can we do? We must be able to understand their needs and to stay with them, protecting the uniqueness of their person in the face of an imminent death.

Active treatment of patients with “incurable disease” is a subject very close to my heart, because I have been dealing with this issue for 28 years, the first 18 of which as a medical oncologist and the last 10 as a palliative doctor in the Hospice of the Casa di Cura Beato Luigi Palazzolo, where I am still working today. This was the first Hospice to be opened in Bergamo in 2000.

Over these 10 years I have cared for 2,200 patients in the last phase of their life, often sitting by their bedside and watching over them as they died.

Dying patients have become teachers for me.

Joan Alifax says that “…death is not usually regarded in contemporary Western culture as a teacher with whom to spend time, but rather as a looming, biological and even moral failure to be denied and avoided…” [1].

I know it is not easy to talk about death: “Whereof one cannot speak, thereof one must be silent.” wrote Ludwig Wittgenstein [2]; but we cannot avoid talking about death; death is the one and only certainty of our lives, our acceptance of death influences not only the experience of dying but also the experience of living, so “We must only talk about what we cannot remain silent about.” as Friedrich Nietzsche said [3]. Death requires both silence and words.

“What to do with the dying?” wonders Christiane Jomain in her book *Mourir dans la tendress* [4]: we have got three choices:

- to remove death—which is impossible, a utopia,
- to abolish the dying—the possibility of euthanasia,
- to accompany the dying—in such a way as to enable them to experience death as the last stage of their growth.

Accompanying the dying in this way may be considered an active treatment.

I have often asked myself what active treatment is and what it means.

I believe that active treatment of patients with incurable disease is not limited to the care of the patients’ symptoms, but goes further.

We can and must control pain, breathlessness, nausea, and vomiting, intestinal obstruction, insomnia… but something else remains for us to face, something paradoxically made more evident by the absence of symptoms: the patients’ emotional suffering, suffering deep in their heart, the most hidden, but which becomes apparent in the season of death.

You may well say, “This is not our problem. We are health care specialists, we are not philosophers or psychologists”, it is a fair point but I want to stress the fact that this too is active treatment of patients with incurable disease.

Helping individuals live their final days in comfort and dignity is one of the most important responsibilities of our profession.

In my opinion, we cannot speak about active treatment if we do not understand that the concept of an incurable disease does not mean that one can no longer care for the patient.

In fact, in palliative care, we adhere to the principal that much can be done when it seems that there is no more we can do.

So what can be done? The answer to this question is provided by the individual patients’ needs: but what are the patients’ needs?

In my experience the needs of the dying coalesce around a series of existential nuclei, of which the most important are the following:

- The first need is for the patients to feel welcome and accepted, whoever they may be; a welcoming acceptance which shows empathy and respect for the person concerned.
- The second need is to have someone listen to them. As Eugenio Borgna says “Listening means trying, sometimes desperately, to understand what lies beneath the patient’s moods, sadness, melancholy and also their moments of joy.
  Listening also means being able to fully comprehend the importance of the spoken language, the language of silence and that of facial expressions, the language of tears and the language of smiles.” [5].

By communicating their suffering in whichever way may be possible for them, the sick people are posing a daunting and lacerating question.

They ask us if they can still consider themselves a person; if they still have the dignity they once did, if their life is still worth living and if, despite their physical transformations, they have still kept their value and their humanity.

This means:

- supporting their choices, thus making the matter of illness and death something which they really feel is their own, experienced very much in an active as opposed to passive way;
- giving them the most important possibility: that of remaining themselves until the last;
- protecting the uniqueness of their person in the face of an imminent death [6].

Here we come to the great problem:

how much of the truth, if any, should we tell the patient?

Cicely Saunders said “We should allow the sick persons to reach their own truth: that which they are able to bear.” [6].

It means that there is no one correct way of doing this, or one best approach that is appropriate for all patients, but each patient needs an individualized approach, depending on the individual circumstances.

In my experience this goal of making the patients the protagonists of their life and their death is often hampered by the lack of clarity and awareness of what is actually happening.

I think many of our patients know they are dying but are waiting for others, doctors and family to tell them. Perhaps they would prefer people with whom they have a special relationship of affection, tenderness, complicity, and love to be the ones to break the news.

Untruth, when people are aware of what is wrong with them, leads to a profound sense of isolation.

I often see this situation: the sick persons know they are dying, but do not want to say so because they are afraid of worrying their relatives.

The relatives do not want to speak to the sick persons about the situation because they in turn are afraid of worrying them.

Doctors and nurses would like to tell the truth, but they cannot. So we have a group of islands each of which is isolated from the others in their own, impenetrable solitude, at a time when the greatest need is for communication and physical contact, a hug in order to let the words and the tears come out.

How many words said at the cemetery could have been said before!

In the book “The Death of Ivan Iljc” L. Tolstoy wrote:

“What tormented Ivan Ilych most was the deception, the lie, which for some reason they all accepted, that he was not dying but was simply ill, and he only need keep quiet and undergo a treatment and then something very good would result. He however knew that do what they would nothing would come of it, only still more agonizing suffering and death. This deception tortured him—their not wishing to admit what they all knew and what he knew, but wanting to lie to him concerning his terrible condition, and wishing and forcing him to participate in that lie. Those lies—lies enacted over him on the eve of his death and destined to degrade this awful, solemn act to the level of their visitings, their curtains, their sturgeon for dinner—were a terrible agony for Ivan Ilych. And strangely enough, many times when they were going through their antics over him he had been within a hairbreadth of calling out to them: ‘Stop lying! You know and I know that I am dying. Then at least stop lying about it!’ But he had never had the spirit to do it.” [7]

The truth, when told in a suitable way, makes things easier for the patient and family.

A recent work of Lunquist et al. [8] shows that “Providing information of imminent death to a patient with cancer at the end of life does not seem to increase pain or anxiety, but it does seem to be associated with improved care and to increase the likelihood of fulfilling the principles of a good death.”

Cicely Saunders reminds us [6]:

- We must learn what this pain is.
- We must learn what it means to feel so sick, to be about to leave this life and its activities, what it means to realize that one is losing one’s physical and mental abilities and that one is going to abandon loved ones and responsibilities.
- We must learn what it means feel close to the patients without feeling like the patients, if we want to be able to listen to them in the way they need and provide the kind of stable support they require to help them find their way.

It is hard to bear the pain of others, which is often so personal, a wound so unique and unrepeatable in its identity that it cannot be fully understood and experienced from the outside.

What can be shared, however, is the question of meaning.

- Why?
- What is the meaning?
- Where does it come from?

How many times I felt lost while watching patients who had become a shadow of the person they once were, no longer able to speak, see, communicate; however, they continued living and I asked myself what it all meant, seeking an answer to their existence in a particular way and light years away from the models proposed by the mass media which continually extol beauty, physical efficiency, independence… What is the point? What do these dying patients want to tell me?

I remember Father David Maria Turoldo, whose life was both song and crying, the song for the believer and the crying for the suffering; he said “There are sorrows for which no words exist in any dictionary. Pain and anguish in front of which the best answer is silence. Neither philosophy nor sermons are needed here, the best remedy (a remedy I say, not an answer) will be our participation as a friend, our loving presence, our “being with” the persons who are suffering … will be compassion and acceptance as far as it is possible, although this should not mean giving up the struggle or no longer seeking deliverance from evil, from all evil, it mustn’t mean a fatalistic resignation, but it may mean patience, which is entirely different.”[9]

“In your patient you’ ll possess your souls” [10].

The Christian answer to the mystery of death and suffering is not an explanation, but a presence.

“Watch with me” Matthew’s Gospel, from 26.36 to 46.

“Watch” means above all, simply, “being here”.

I remember Mary: she was 60 years old and still beautiful, but a devastating gastric cancer was destroying her. She knew her situation. That afternoon I had to give her yet more bad news.

I went into her room not knowing what to say… we sat on her bed and looked out of the window, it was a beautiful spring day, the sky was blue and the wind stirred the branches of the birches in front of the clinic.

I was there with her for half an hour, without telling her anything, she had already figured everything out…, then, I hugged her and went out of her room.

I can remember that afternoon as if it was yesterday and yet, 19 years have passed. It remains the best experience of communication I have had in the whole of my professional life.

“Staying means not running away, it means staying with someone despite the deep discomfort the pain and suffering of the others can cause in us.

Because for those who suffer, to feel the fact they are not alone in this desperate time, opens the doors to hope (a solution that can still come)…” as Mertens says [11].

I remember Alice: she was 35 years old and had an invasive liver cancer, she was dying. I had not seen her for 4 days because I had been at a medical conference.

I went into her room that night, Alice was devastated and had not spoken for three days. I remember that I stroked her forehead and said: “I’m back, I am here with you.”

Alice told me with great difficulty: “I’ve been waiting for you”, then she smiled and died in my arms.

Julia was 14 years old and had a terrible sarcoma. She died last August, after 2 years of struggle against the disease.

Julia was able to transform these 2 years in a hymn to life; she said:

“The fact is that people are afraid of disease and suffering. There are many sick people who are alone, all their friends disappear, frightened. Don’t be afraid! If the others stay with us, are close to us, put their hand on our shoulder and tell us ‘Come on, you can make it!’, we can have the strength to go forward. If this does not happen, you wonder: Why are they going so far away? If they are afraid, then I am afraid too…. Why should I fight to get better if no one remains close to me?”.

Marco was 18 years old and had an incurable lymphoma. Every evening, before going home, I passed by to ask how he was.

One evening I asked him what his greatest dream was, he replied: “To become a fisherman”.

“If you want to become a fisherman, you must have a boat and you have to give it a name: what will you call it?” I asked.

“I’ll name my boat after you” he answered.

- Active treatment can mean allowing the patient to continue to dream, because even if the body is paralyzed in a bed, the mind can go wherever it wants, can fly over the highest peaks and glide over the greenest plains, dive into the sea and be lulled by the waves.

After a few days Marco began to understand that he was dying, so he told me that he wanted to die on the beach in Marsala, his home town, a small town in Sicily. We were able to make his wish possible. Marco died by the sea that he loved so early.

We must not delude the patient, but we must not take away their hope!

- Active treatment means not destroying hope.

What is hope?

Emily Dickinson said [12].

*“Hope” is the thing with feathers—*

*That perches in the soul*—

*And sings the tune without the words*—

*And never stops*—*at all*—

*And sweetest*—*in the Gale*—*is heard*—

*And sore must be the storm*—

*That could abash the little Bird*

*That kept so many warm*—

*I’ve heard it in the chilliest land*—

*And on the strangest Sea*—

*Yet, never, in Extremity,*

*It asked a crumb*—*of Me.*

Hope is a theological virtue.

Here we are speaking of the hope which does not abandon, while recognizing the gravity of the situation.

True hope gives the confidence that nothing is ever really lost, and that following the deprivation, for a natural and divine law, a greater gift will come.

But how and what to hope in the hour of death?

Perhaps an answer to this question is the definition of health proposed by Pope John Paul II°:

*Health is a dynamic tension towards harmony* [12].

If this is true disease is not darkness, it is not the night, but a stage in this dynamic tension towards harmony, which for us Catholics is the Resurrection.

Could this be our hope?

Palliative care medicine is and remains a health service. So not a medicine for the dying and for aiding death, but a medicine for men, who remain living until their death (Spinsanti) [13].

The experience of palliative care has an impact that makes it impossible to accept the request for euthanasia.

- Active treatment means giving up on euthanasia but also on aggressive medical treatment.

In means standing in the middle, in order to protect the best possible quality of life of patients and their families.

It means preventing confusion: palliative sedation, pain relief, not starting disproportionate treatments, interruption of disproportionate treatments, are not acts of euthanasia but professionally correct gestures.

In a recent work of Saito et al. [15] we can read:

“…Continuing chemotherapy for advanced NSCLC until very near death is associated with a decreased likelihood of receiving hospice care but not prolonged survival. Oncologists should strive to discontinue chemotherapy as death approaches and encourage patients to enroll in hospice for better end-of-life palliative care…”

- Active treatment is fundamental not only for patients but also for caregivers.

To do this work we need to focus attention on our own spiritual resources because, in my opinion, we cannot give what we have not got inside us.

Here I have not time to speak about spirituality, which would require an entire medconference, but I would like to stress the fact that an attitude of openness and inclusiveness is essential as a basis for working with the dying, death, caregivers, and the grieving.

“It takes a big heart to hold so much suffering” said Joan Alifax, “It takes a heart as wide as the world” said Sharon Salzberg [1].

Now, let me give you one last challenge:

from Mattheuw’ s Gospel 25, 34–36 [16].

“…Then the King will say to those at his right hand, `Come, O blessed of my Father, inherit the kingdom prepared for you from the foundation of the world; for I was hungry and you gave me food, I was thirsty and you gave me drink, I was a stranger and you welcomed me, I was naked and you clothed me, I was sick and you visited me, I was in prison and you came to me…”.

One is left breathless reading these words: they turn everything completely upside down both fear and perplexities, and they give the dying sick persons an enormous dignity, not only because it means they will continue to be that person right up until their last breath, deserving therefore care and love, but above all because sick persons become an instrument of redemption, of my redemption, of my salvation, of the salvation of each one of us. If this is true than the temptations regarding euthanasia disappear because one cannot suppress an occasion for love and salvation, instead one must come alongside it, on tip-toe, one one’s knees, with one’s heart overflowing with gratitude.

*The great dignity of the dying is to remember in every moment that He was here, He is still here and His presence gives redemption* [6].

Only with love, faith and hope can we conquer death. In this way we can get to the root of all life and have the firm conviction that only love is credible.
